# Evolution of Learning Styles in Surgery Comparing Residents and Teachers: Cross-Sectional Study

**DOI:** 10.2196/64767

**Published:** 2025-05-08

**Authors:** Gabriela Gouvea Silva, Carlos Dario da Silva Costa, Bruno Cardoso Gonçalves, Luiz Vianney Saldanha Cidrão Nunes, Emerson Roberto dos Santos, Natalia Almeida de Arnaldo Rodriguez Castro, Alba Regina de Abreu Lima, Vânia Maria Sabadoto Brienze, Antônio Hélio Oliani, Júlio César André

**Affiliations:** 1 Center for Studies and Development of Health Education, Faculdade de Medicina de São José do Rio Preto, Avenida Brigadeiro Faria Lima, 5416, São José do Rio Preto, 15090000, Brazil, 55 17982022252

**Keywords:** learning, general surgery, medical education, internship and residency, surgeons, Brazil

## Abstract

**Background:**

Studies confirm a relationship between learning style and medical career choice in the learning style patterns observed in distinct types of residency programs. Such patterns can also be applied to general surgery, from medical school to the latest stages of training. Aligning teaching strategies with the predominant learning styles in surgical residency programs has the potential to make training more effective.

**Objective:**

This study aimed to determine the learning styles of general surgery residents and professors in a Brazilian teaching hospital and compare the results with the existing literature.

**Methods:**

This was a cross-sectional study conducted in a teaching hospital of a public university in Brazil. Thirty-four general surgery residents of any year of training and 30 professors participated in the study. Participants completed a sociodemographic survey and David Kolb’s Learning Style Inventory. This was used to classify participants into one of four distinct types of learners: accommodating, diverging, assimilating, and converging. The relationship between sociodemographic data and learning styles was analyzed using the Fisher test, adjusted using the Bonferroni method, and the effect size was measured using the Cramer V test.

**Results:**

The learning style distribution was similar in both groups, with 43,75% diverging, 42,18% accommodating, 10.93% assimilating, and 3.12% converging styles. A significant relationship was found between sex and learning style (*P*=.049) and between age and learning style for professors (*P*=.029). The effect sizes were strong (0.46) and very strong (0.506).

**Conclusions:**

The prevalence of learning styles among general surgery residents and professors at this Brazilian hospital differs from that observed in previous studies, with more diverging and accommodating learners and fewer converging learners, suggesting a shift in learning styles. Understanding learning styles is important for effective surgical training programs. Further research with larger and more diverse populations is needed to confirm these results and explore the factors contributing to the observed differences in learning styles.

## Introduction

### Background

The concept of learning styles was first developed at the beginning of 1960 as a result of the interest in individual differences while learning [1]. According to Dunn [2], everyone has a unique learning style, like a signature. In this prospect, adjusting the teaching to the different learning styles may help learners and improve educational outcomes. In the current literature, there are various models to determine the learning styles. There is a long and active discussion about whether learning styles are fixed or flexible, and to what extent the context can determine it [3]. Adapting learning styles can enhance student engagement, motivation, and academic performance [4]. Furthermore, the integration of technology and personalized learning approaches has shown promise in enhancing medical education [5].

To provide a more comprehensive understanding, learning styles can be defined as the cognitive, affective, and physiological traits that serve as relatively stable indicators of how learners perceive, interact with, and respond to the learning environment [6]. Empirical evidence supports the existence of learning styles, demonstrating that individuals exhibit consistent preferences and strengths in how they approach learning tasks. For example, some learners may excel in visual tasks, while others thrive in auditory or kinesthetic activities [7].

The Kolb Experiential Learning Theory (ELT) is a prominent framework for understanding learning styles. The ELT posits that learning is a cyclical process involving four modes: concrete experience (CE), reflective observation (RO), abstract conceptualization (AC), and active experimentation (AE) [8]. Individuals develop preferences for certain modes, leading to four distinct learning styles: converging (AC/AE), diverging (CE/RO), assimilating (AC/RO), and accommodating (CE/AE). The Kolb Learning Style Inventory (LSI) is a widely used tool for assessing these preferences. The validity of Kolb’s work in the context of medical education has been demonstrated in numerous studies [9-13], which have found that medical students and professionals exhibit distinct learning style preferences that can influence their academic and professional performance.

Knowledge is the main domain of medical education, but outcomes strongly depend on other domains such as attitude, lifelong learning, and empathy; in surgery, some domains are central including resilience, craftsmanship, and decision-making, among other domains [14].

### Research Gap and Problem Statement

Despite the established importance of learning styles in education, limited research has specifically examined their prevalence and impact within general surgery residency programs, particularly in diverse cultural and geographical settings. The clinical and surgical environments present unique challenges for both trainees and educators, requiring the development of complex skills and behaviors [14]. Understanding how surgical residents learn is crucial for optimizing the training process and ensuring the development of competent and well-rounded surgeons.

Moreover, current surgical trainees come from diverse educational, cultural, ethnic, and gender backgrounds, and personal factors also influence their learning characteristics [15]. Little is known about the teaching and learning preferences among surgeons and how they influence the effectiveness of training [16]. Addressing this gap in knowledge is essential for designing effective and inclusive surgical training programs that cater to the diverse needs of learners. To this end, simulation-based surgical training has emerged as a valuable tool for enhancing technical skills and improving patient outcomes [17].

### Research Aims and Objectives

Little is known about the teaching and learning preferences among surgeons and how they influence the effectiveness of training [16]. Despite its relevance, studies investigating learning styles in the context of general surgery residency are scarce, especially in countries outside North America and Europe.

Therefore, to address the gaps in understanding learning styles in general surgery, particularly in diverse cultural and geographical settings, this study aims to (1) determine the learning styles of general surgery residents and professors in a Brazilian teaching hospital; (2) compare these findings with existing literature on learning styles in surgery; and (3) discuss the implications of these findings for surgical training programs.

By providing data from a Brazilian teaching hospital, we aim to contribute to a more comprehensive understanding of learning styles in surgical training and inform the development of more effective and inclusive surgical education strategies. This knowledge can inform the development of more effective and inclusive surgical education strategies, ultimately leading to better-prepared and more competent surgeons.

## Methods

### Study Design and Setting

This cross-sectional study was conducted in 2022 at the Hospital de Base de São José do Rio Preto, a teaching hospital affiliated with Faculdade de Medicina de São José do Rio Preto (a public university in São Paulo, Brazil).

### Participants and Recruitment

The study population consisted of general surgery residents in any year of training and hospital professors. All participants were over 18 years old and signed the free and informed consent form.

### Data Collection

Data collection involved two instruments: a sociodemographic survey and David Kolb’s LSI. The sociodemographic survey collected information on participants’ age and sex, and years of residency (for residents) or teaching experience (for professors). The LSI is a validated tool that consists of 12 questions, each with four statements that the participants ranked from 1 to 4 according to their learning preferences. The LSI tool classifies the participants into one of four types of learners based on Kolb’s learning cycle: (1) accommodating (learn primarily by experience), (2) diverging (learn by RO), (3) assimilating (learn by exploring associations and interrelationships), and (4) converging (learn by doing or trying things with practical results) [18].

The LSI test was administered in a controlled environment, with a researcher present to provide instructions and clarify any doubts. Participants had 30 minutes to complete it. The sociodemographic survey was completed immediately after completing the LSI test.

### Statistical Analysis

#### Software

Data analysis was performed using the Statistical Package for the Social Sciences (SPSS), version 26.0 (IBM Corp.).

#### Normality Check

Due to the relatively small sample size and the nature of the data, the Shapiro-Wilk test was used to assess the normality of continuous variables (age and years of experience).

#### Statistical Tests

A *P* value <.05 was considered statistically significant. The relationship between data was calculated using the Fisher test, adjusted by the Bonferroni method [19]. The Fisher exact test was chosen due to the small sample size and the presence of categories with expected frequencies lower than 5 [20]. The size effect was measured using Cramer V test, which indicates the grade of association between variables: the result is stronger as it approaches the value of 1 [21].

#### Power

The sample size was calculated using the formula for finite populations, considering a confidence level of 95%, a margin of error of 5%, and an expected prevalence of 50% for each learning style. The minimum sample size was 67 participants, and the total number of residents and professors was 80 [22].

#### Data Exclusion

Questionnaires that were responded to incorrectly according to Kolb’s rules were discarded.

### Ethical Considerations

The study was approved by the Research Ethics Committee of Faculdade de Medicina de São José do Rio Preto (approval number: 12345/2022). All participants signed the free informed consent form. Data were anonymized.

### Recruitment

This study is grounded in Kolb’s ELT, which posits that learning is a cyclical process involving four modes: CE, RO, AC, and AE [23]. Individuals develop preferences for certain modes, leading to four distinct learning styles:

- Converging: Individuals with this learning style excel in AC and AE. They are practical, enjoy problem-solving, and are skilled at applying theories to real-world situations.
- Diverging: Individuals with this learning style excel in concrete CE and RO. They are imaginative, enjoy brainstorming, and are skilled at generating ideas.
- Assimilating: Individuals with this learning style excel in AC and RO. They are logical, enjoy analyzing data, and are skilled at creating models and theories.
- Accommodating: Individuals with this learning style excel in concrete CE and AE. They are hands-on, enjoy taking risks, and are skilled at implementing plans and getting things done.

Our logic model is based on the premise that aligning teaching strategies with the predominant learning styles of surgical residents and professors can enhance the effectiveness of surgical training. We hypothesize that by identifying the learning styles of our participants and tailoring instructional approaches accordingly, we can improve learning outcomes and promote a more engaging and inclusive learning environment.

Table 1 provides a more detailed overview of the four learning styles, including concrete examples of learning activities and instructional approaches that are best suited for each style.

**Table 1.** Learning styles, characteristics, and instructional approaches. Source: [8].

Learning style	Characteristics	Example learning activities	Example instructional approaches
Converging	Practical, problem-solver, applies theories	Simulation-based training, case studies	Problem-based learning, hands-on workshops
Diverging	Imaginative, brainstormer, generates ideas	Group discussions, reflective writing	Mentoring, collaborative projects
Assimilating	Logical, analytical, creates models	Literature reviews, data analysis	Lectures, seminars
Accommodating	Hands-on, risk-taker, implements plans	Surgical procedures, clinical rotations	Apprenticeship, on-the-job training

All general surgery residents were invited to answer printed free and informed consent form and the LSI’s test, in person. The same was done with the faculty members. The questionnaires were then collected and transformed into digital archives, processed in digital tables after codification.

### Statistical Analysis

#### Power

The sample size was calculated using the formula for finite populations, considering a confidence level of 95%, a margin of error of 5%, and an expected prevalence of 50% for each learning style. The minimum sample size was 67 participants, and the total number of residents and professors was 80 [22].

## Results

A total of 64 participants (34 residents and 30 professors) were included in this study. The sociodemographic characteristics of the participants are presented in Table 2. Among the 34 residents, 18 (52.9%) were male and 16 (47.1%) were female. Most residents (91.2%, 31/34) were under 30 years of age. Among the 30 professors, 24 (80%) were male, and 6 (20%) were female, and most of them (17/30, 56.7%) were between 40 and 70 years of age. All professors graduated from universities when traditional teaching methods (ie, primarily lecture-based instruction with limited student interaction) were used, whereas 47% of the residents graduated from universities that used active or mixed teaching methods (ie, incorporating strategies such as problem-based learning, group work, and case studies to promote student engagement).

**Table 2.** Sociodemographic characteristics of participants.

Characteristics	Residents (n=34) N (%)	Professors (n=30) N (%)
Age (years)		
<30	31 (91.2)	2 (6.7)
30‐39	3 (8.8)	11 (36.7)
40‐70	0 (0)	17 (56.7)
Sex		
Male	18 (52.9)	24 (80)
Female	16 (47.1)	6 (20)
Teaching method used at the University of origin		
Traditional	18 (52.9)	30 (100)
Active or mixed	16 (47.1)	0 (0)

The distribution of Kolb’s learning styles is presented in Table 3 and Multimedia Appendix 1. The most prevalent learning styles were diverging (18/34) in the residents’ group and accommodating (17/30) in the professors’ group.

**Table 3.** Learning styles among surgery groups.

Learning styles	Residents N (%)	Professors N (%)	Total N (%)
Converging	1/34 (2.94)	1/30 (3.33)	2/64 (3.12)
Assimilating	5/34 (14.7)	2/30 (6.7)	7/64 (10.93)
Accommodating	10/34 (29.4)	17/30 (56.7)	27/64 (42.18)
Diverging	18/34 (52.9)	10/30 (33.3)	28/64 (43.75)

The relationship between sociodemographic data and learning styles was analyzed using the Fisher exact test (Table 4). A significant association was found between sex and learning style (*P*=.049; Cramer V=0.46), indicating a strong effect size. However, determining which specific categories were significantly different using the Bonferroni post-hoc test was not possible. Among professors, a significant relationship was observed between age and learning style (*P*=.029; Cramer V=0.506), suggesting a very strong effect size. However, specific age groups that differed significantly could not be identified with the Bonferroni post-hoc test, possibly due to the small sample size.

**Table 4.** Relationship between sociodemographic data and learning styles.

Variables	*P* value (Fisher exact test)	Effect sizes (Cramer V)
Sex	0.049^a^	0.46 (strong)
Age (residents)	0.999	0.12 (weak)
Age (professors)	0.029^a^	0.506 (very strong)
Teaching method used at the university of origin (residents)	0.999	0.08 (weak)

a Statistically significant at P<.05

## Discussion

### Principal Findings

Our study, utilizing Kolb’s LSI, identified the distribution of four learning styles among general surgery residents and professors at a Brazilian teaching hospital. These learning styles are:

- Diverging: Learners who excel in CE and RO, are imaginative, and generate ideas effectively.
- Accommodating: Learners who excel in CE and AE, are hands-on, and enjoy implementing plans.
- Assimilating: Learners who excel in AC and RO, are logical, and create models and theories.
- Converging: Learners who excel in AC and AE, are practical, and apply theories to real-world situations.

The most prevalent learning styles were diverging (52.9%) in the residents’ group and accommodating (56.7%) in the professors’ group (Table 2 and Multimedia Appendix 1). A significant association was found between sex and learning style (*P*=.049; Cramer V=0.46), indicating a strong effect size. Among professors, a significant relationship was observed between age and learning style (*P*=0.029; Cramer V=0.506), suggesting a very strong effect size.

Table 2 and Multimedia Appendix 1 show that while diverging was the most common style among residents and accommodating was most common among professors, the overall learning style distribution was relatively similar between the two groups. This convergence, where both residents and professors exhibit a blend of diverging and accommodating tendencies, can potentially facilitate both teaching and learning [16]. The shared presence of these styles suggests that both groups may value CEs and RO (diverging) as well as hands-on activities and practical application (accommodating).

This alignment can be leveraged to support instruction in different ways. For diverging learners (both residents and some professors), emphasize group discussions, brainstorming sessions, and reflective writing assignments. Encourage the sharing of diverse perspectives and the exploration of different approaches to surgical problems. In contrast, for accommodating learners (both professors and some residents), Provide opportunities for hands-on practice, simulation-based training, and real-world clinical rotations. Encourage AE and problem-solving in practical settings. By incorporating these strategies, educators can create a learning environment that caters to the predominant learning styles of both residents and professors, fostering more effective communication, engagement, and knowledge acquisition.

However, it is important to acknowledge that the similarity in distribution does not guarantee a perfect match for all individuals. The relatively lower prevalence of converging and assimilating styles in both groups suggests that those learners might require more tailored support and learning opportunities to ensure their needs are met. This underscores the importance of mapping learning styles when designing a comprehensive residency program, as it provides a basis for guiding the learning needs of all residents and professors, not just the majority.

### Implications of Findings

The findings of our study have important implications for surgical education. Understanding the predominant learning styles of residents and professors can help adapt teaching strategies and curriculum design to better meet their needs. For example, incorporating more RO and practical experiences can benefit diverging and accommodating learners while also providing opportunities for AC and AE to support assimilating and converging learners.

Furthermore, with the occurrence of the pandemic, the increased distances imposed by contact restrictions have further deepened these changes. The COVID-19 pandemic has also presented unique challenges to surgical training, with restrictions on in-person learning and clinical experiences. A pan-Romanian survey by Moldovan et al [24] highlighted the impact of the pandemic on orthopedic residents, including reduced surgical volume, limited access to educational resources, and increased stress and anxiety. These challenges may have further influenced the learning styles and preferences of surgical residents and professors during this period.

### Comparison With Prior Work

Few studies on learning styles in surgery were found in the literature, but we can state that our results are different from previous results.

In the 1980s, Baker III et al [25] reported a prevalence of converging (46%), followed by accommodating (26%) and assimilating (20%) styles among surgeons. In the 2000s, this pattern was confirmed by Contessa et al [26]. They argued that surgical practice requires quick decision-making and problem-resolution, justifying the converging style and its more pragmatic view. In 2007, Mammen et al [27] published similar results obtained in the US population.

After Quillin [28] reduced his working hours in general surgery residency, he showed the results collected from 1999 to 2012. At that time, the proportion of accommodating learners was higher, especially after 2003, when the workload was reduced [28].

In 2017, for the first time, diverging learners became the majority in a study with 47 surgeons in the United Kingdom [29]. In 2018, also in the United Kingdom, a study with residents in various surgical areas found that converging, followed by accommodating styles were predominant [30]. In 2020, similar results were published in Scotland by Hopkins et al [15]. The most recent publication on the topic reported a predominance of assimilating followed by converging styles in Spain [31]. Table 5 and Multimedia Appendix 2 show the existing literature.

**Table 5.** Data of learning styles in surgery through time around the world.

Author	Publication year	Country	Population (n)	Diverging	Accomodator	Assimilating	Converging
Baker III	1985	USA	Surgeons (39)	8%	26%	20%	46%
Drew	1999	UK	Basic surgical trainees (52)	3.9%	27%	9.6%	59.5%
Mammen	2007	USA	General surgery residents (91)	10.6%	14.6%	17.2%	57.8%
Brown	2018	UK	Medical students (60)	20.8%	30.2%	17%	32%
Parra	2021	Spain	Surgical residents and staff (64)	14.1%	21.9%	39.1%	25%

The results of the present study were diverging (43,8%), accommodating (42,2%), assimilating (11,0%), and converging (3,12%) styles. These results amplify the existing literature, showing an increase in diverging and a decrease in converging styles over the years. These findings indicate a shift in the learning preferences of surgical residents and professors, which may have been influenced by various factors, such as changes in surgical education, technological advancements, and sociocultural aspects.

The geographical location may be a possible explanation for our results, as previous studies were conducted in North America and Europe (Multimedia Appendix 1). Cultural differences and variations in surgical training programs across countries may have contributed to the observed differences in learning styles. Another hypothesis may be the course of time: the last two decades have seen huge technological changes, when social media, smartphones, and laptops became widely available, greatly impacting the teaching-learning process [32]. Recent studies have further explored the impact of digital technologies on medical education, highlighting both the opportunities and challenges associated with their integration [5]. Furthermore, with the occurrence of the pandemic, the increased distances imposed by contact restrictions have further deepened these changes [33].

The differing proportions of female residents (47.1%) and professors (16.0%) highlight the ongoing evolution of gender representation in surgery. The historical underrepresentation of women in surgical fields may contribute to differences in observed learning styles between residents and professors [34]. Despite this, the increasing participation of women in surgery over recent decades is a positive trend [22].

### Strengths and Limitations

The population included is a small sample of a larger Brazilian surgical group. More data can be further collected to compare the country with other nations, in America, Europe or even Asia. The medical reality in Brazil is diverse and worth a broader approach.

In addition to the small sample size, our study has several other limitations that should be acknowledged. The study had a sampling bias. Our sample was drawn from a single teaching hospital in Brazil, which may not be representative of all general surgery residents and professors in Brazil or other countries. This limits the generalizability of our findings. Additionally, the voluntary nature of participation may have introduced selection bias, as those who chose to participate may differ systematically from those who did not. In addition, there was measurement bias; the Kolb LSI is a self-report instrument, which is subject to social desirability bias and response bias. Participants may have answered the questions in a way that they perceived as more favorable or aligned with societal expectations, rather than reflecting their true learning preferences. Moreover, our study did not fully explore the potential influence of various sociodemographic factors, such as cultural background, socioeconomic status, and prior educational experiences, on learning styles. However, we did not collect data on other potentially relevant sociodemographic factors such as ethnicity, social class, migration background, or detailed information about prior educational experiences. These factors may interact with learning styles in complex ways and could have influenced our results.Finally, the cross-sectional design of our study limits our ability to draw causal inferences about the relationship between learning styles and other variables. A longitudinal study would be needed to examine how learning styles evolve over time and how they impact training outcomes. These limitations should be considered when interpreting our findings.

Further research is needed to explore the underlying factors that influence these learning styles, such as personality traits, prior educational experiences, and cultural background. Understanding these factors could allow for more tailored interventions to optimize learning. Moreover, future studies should investigate the potential impact of different learning styles on surgical performance metrics, such as technical skill acquisition, error rates, and patient outcomes. This would provide valuable insights into how learning style preferences translate into real-world surgical practice.

### Conclusions

This study found that diverging and accommodating learning styles were more prevalent among general surgery residents and professors in a Brazilian university hospital, differing from previous North American and European studies. The decreased prevalence of the converging style is notable and may be due to changes in surgical education, technology, and cultural differences. Understanding these learning styles can guide more effective and inclusive teaching strategies in surgical residency programs. Further research with larger, diverse populations is needed to explore the relationships between learning styles, demographics, and training outcomes.

## Supplementary material

10.2196/64767 Multimedia Appendix 1 Learning styles prevalent in surgery groups.

10.2196/64767 Multimedia Appendix 2 Timeline of surgical learning styles according to the existent literature.

## Abbreviations

AC: abstract conceptualization
AE: active experimentation
CE: concrete experience
ELT: Experiential Learning Theory
LSI: learning style inventory
RO: reflective observation
