# Supplementary figures and images for: Evolution of Learning Styles in Surgery Comparing Residents and Teachers: Cross-Sectional Study
Source: JMIR Med Educ. 2025 May 8;11:e64767. doi: 10.2196/64767 (PMC12080965; doi:10.2196/64767)

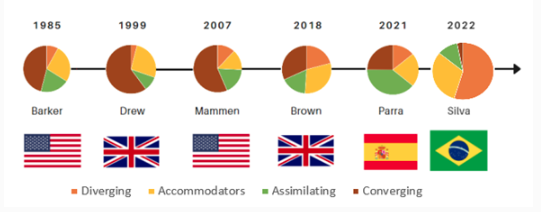

Supplement: Multimedia Appendix 1 [file mededu-v11-e64767-s001.png]

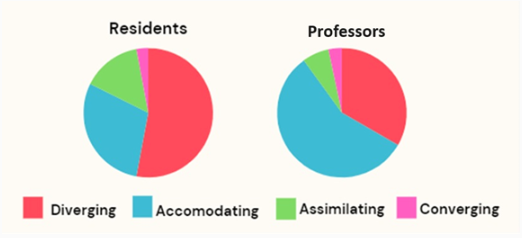

Supplement: Multimedia Appendix 2 [file mededu-v11-e64767-s002.png]
